# When the Uterus Competes for Perfusion: Management of a Pregnant Patient with Bypass Graft Occlusion

**DOI:** 10.1155/2019/2432809

**Published:** 2019-10-07

**Authors:** Sarah McGriff, Paige Percer, Iberia Sosa, Hector Mendez-Figueroa, Joseph L. Mills, Manisha Gandhi

**Affiliations:** Baylor College of Medicine, Texas Children's Hospital, Houston, Texas, USA

## Abstract

**Background:**

Peripheral arterial disease (PAD) in pregnancy has serious implications and requires multidisciplinary management. This becomes even more complicated in the setting of active disease and history of prior vascular grafts.

**Case:**

A woman presented with increasing left lower extremity pain at 18 weeks of gestation with a complex history of PAD and a previous bifurcated aorta-left femoral, -right iliac bypass. CT angiogram demonstrated known occluded bypass graft. A multidisciplinary team of providers developed guidelines for potential surgical intervention based upon clinical symptoms.

**Conclusion:**

Pelvic PAD can worsen in pregnancy in the setting of the enlarging uterus, which can potentially deplete perfusion of existing collateral vessels. Symptomatic approach to worsening disease provided an effective management strategy in this case.

## 1. Introduction

Pregnancy following aortic bypass or replacement grafting is uncommon, but has been previously reported [1–6]. Bypass grafts for arterial occlusive disease are often placed in an older population beyond typical child-bearing age. Overall, maternal and neonatal outcomes are favorable with careful management. A search of the literature revealed one case with expectant management of claudication with no permanent sequelae [4], and a single case of graft occlusion during pregnancy [5]. However, the patient with graft occlusion only had symptoms of claudication with physical activity and was successfully managed with antiplatelet therapy using aspirin. We present the management of a pregnancy in a patient with significant peripheral arterial disease (PAD) and an occluded graft from a previous aortic reconstruction that had severe rest pain and disability requiring increased anticoagulation.

## 2. Case

**Figure 1:**
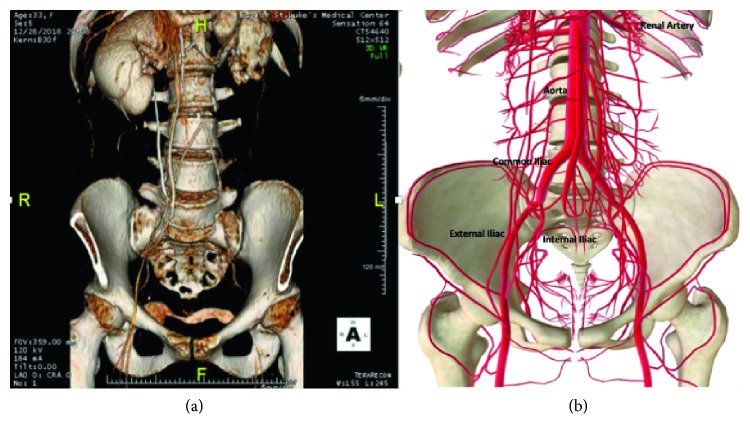
Computed tomography angiogram of the abdomen, pelvis, and lower extremities with runoff (a). Computer generated pelvic vasculature demonstrating the stark difference (b).

The patient is a 33-year-old woman gravida 5, para 3, abortion 1 with chronic hypertension and 3-year history of arterial thrombosis. At age 30, she underwent aortoiliac thrombectomy and bilateral femoral thrombectomies with repair, and was started on warfarin postoperatively. Fifteen months later, noncompliant with anticoagulation, she presented for severe back pain radiating down her legs bilaterally. A repeat CT angiogram revealed a left common iliac artery occlusion with thrombus in the infrarenal abdominal aorta with possible dissection flap above the bifurcation. She underwent aorta-left femoral and aorta-right iliac bypass with a bifurcated polytetrafluorethylene (PTFE) graft (Figure 1). Repeat hypercoagulable testing was negative, and she was discharged on warfarin treatment. The patient continued anticoagulation inconsistently. She presented multiple times over the following year complaining of lower extremity pain. Repeat CT angiograms demonstrated complete occlusion of both arms of the graft with normal runoff and patent pelvic collaterals.

Three years after her initial intervention, she was pregnant and established care with our maternal-fetal medicine practice at 13 weeks of gestation. Her obstetrical history included one prior spontaneous abortion, two term vaginal deliveries, and most recently a cesarean delivery at gestational week 28 due to preeclampsia with severe features. All previous pregnancies occurred prior to aortic reconstruction, and there was no history of thrombotic disease with any pregnancy. The risk of the patient's gestation was reviewed in detail and included discussion of her grafts, disease burden, and anticoagulation requirements. She was offered termination of pregnancy but declined. She was prescribed therapeutic dose enoxaparin (1 mg/kg twice daily) and aspirin 81 mg daily for anticoagulation.

At 18 weeks of gestation, she presented with left leg pain and decreased sensation in the left lower extremity. Repeat CT angiogram showed no interval changes with apparent diversion of some collateral flow to the pregnant uterus. Urine drug screen was positive for cocaine. Due to worsening symptoms likely secondary to increased vascular demand of the gravid uterus and cocaine use, the patient was admitted and started on an unfractionated (UFH) heparin infusion.

After consulting vascular surgery, urgent surgical intervention was not offered as she had conserved sensation and motor function in lower extremities, there was increased likelihood that the gravid uterus would be a barrier to aortic reconstruction, and her occlusive disease appeared stable on repeat imaging. The vascular surgeons further assessed and recommended intervention only if worsening of blood flow, as defined by the loss of the patient's motor function in her lower extremities. If she reached that point, she would require axillofemoral bypass surgery. The goals of care were discussed, and the patient reported she was prepared to lose her leg before jeopardizing the pregnancy. She was discharged on therapeutic enoxaparin at 1 mg/kg twice daily and anti-factor Xa levels were followed monthly to adjust dose accordingly. Throughout the pregnancy, the patient was evaluated multiple times for increasing pain and required supportive care with wheelchair assistance and pain medications.

The patient presented at 31 weeks and 4 days of gestation with superimposed pre-eclampsia with severe features based on requirement of acute antihypertensives and persistent severe headache. Her CT head imaging was negative and she was transitioned to UFH. Her blood pressures stabilized and the headache resolved, but three days later the patient complained of a persistent headache refractory to medications and required increased dosing of her antihypertensive medications. Plans were made for delivery via repeat cesarean delivery. UFH was stopped 6 hours prior to delivery. The patient underwent an uncomplicated repeat cesarean delivery with bilateral tubal ligation with an estimated blood loss of 600 cc. She was managed in the intensive care unit in the immediate post-operative period and restarted on enoxaparin 1 mg/kg BID at 8 hours after delivery. The neonate weighed 1870 g with Apgar scores of 8 and 9 at 1 and 5 minutes, respectively.

**Figure 2:**
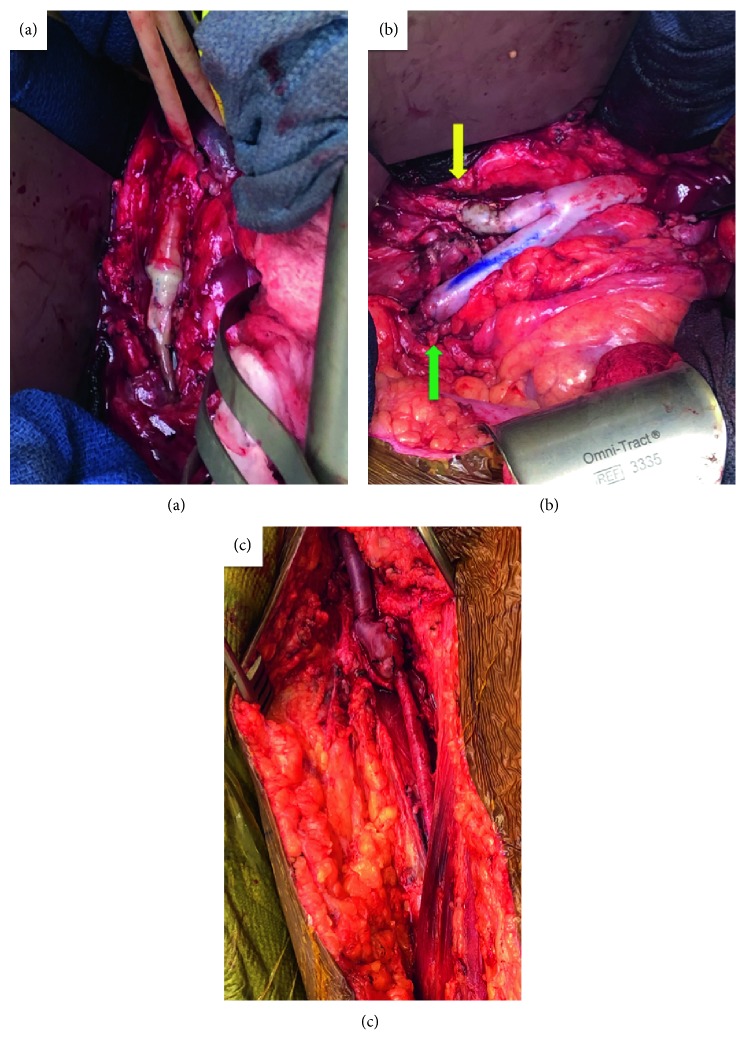
(a) Exposure of the infrarenal aorta and occluded PTFE graft. (b) Cryopreserved aorto-right common iliac allograft (yellow arrow) with left limb anastomosed to an autologous left femoral vein conduit (green arrow) to extend to left common femoral artery. (c) Left femoral vein conduit to left femoral bifurcation anastomosis.

Postoperatively, the patient did complain of some worsening left lower extremity pain, which was evaluated by vascular surgery. However, they determined there was no need for acute surgical intervention as the patient had preserved motor function and her pain was adequately controlled. She was discharged home on enoxaparin 100 mg BID and aspirin 81 g daily. Despite anticoagulation, she was readmitted 11 weeks after delivery with flank pain and found to have a left renal artery infarct and extension of the infrarenal aortic occlusion up to the level of the renal arteries. She was acutely treated with intravenous UFH. Her flank pain resolved and renal function remained within normal limits. However, her left leg pain progressively worsened and required an open surgical juxtarenal aortic thrombectomy and redo aorta-right common iliac,-left common femoral reconstruction with a bifurcated cryopreserved allograft spliced to an autogenous left femoral vein conduit (Figure 2). Prosthetic conduits were avoided because of her presumed hypercoagulable state and failure of previous prosthetic reconstruction.

## 3. Discussion

In this pregnant patient with complex arterial occlusive disease, we were able to successfully manage the pregnancy and the peripheral vascular disease with a multidisciplinary approach. Given that surgical management was more complicated and less definitive due to her gravid uterus, the assessment of motor function was key. This motor function assessment gave our team objective data to assess her disease burden. In regard to the etiology of this patient's peripheral artery disease, we do not have a definitive answer, but suspect cocaine use as potential cause. There is evidence in the literature of limb ischemia and thrombosis in direct association with chronic cocaine use [7, 8].

No specific guidelines exist for maintaining the patency of vascular grafts in the obstetric population. Pregnancy is a known prothrombotic state and often exacerbates hypercoagulable disorders. Even with appropriate anticoagulation, the increased thrombotic risk may still not be mitigated. Multiple international obstetric guidelines recommend that women who have had a prior thrombotic event are prescribed prophylactic heparin throughout their pregnancy and at least 6 weeks postpartum [9, 10]. LMWH is recommended over unfractionated heparin (UFH) given its high bioavailability and ease of administration [11]. We generalized these recommendations to our patient, as any further arterial thrombi in the pelvic or collateral vasculature could result in devastating loss of limb or fetus.

The American College of Cardiology (ACC) and American Heart Association (AHA) developed guidelines for the management of patients with peripheral vascular disease, including revascularization [12]. However, these guidelines contain recommendations that might pose risk to the obstetrical population. Medications aimed at reducing cardiovascular risk factors or maintaining graft patency often have unknown effects or are contraindicated in pregnancy. Of the recommended medications, we prescribed aspirin as a secondary prophylaxis to our patient because it has been shown to prevent arterial thrombosis [12, 13]. Low dose aspirin was additionally indicated due to preeclampsia in her previous pregnancy [14].

Furthermore, surgeries that are recommended by the ACC/AHA for revascularization of aortoiliac occlusive disease are difficult to execute in pregnancy. The optimal timing for surgical intervention in pregnant women is during the second trimester, as the risk posed by anesthesia and surgery is the lowest. However, a challenge with concomitant aortic disease, is that the gravid uterus is too large and blocks the surgical site. According to the ACC/AHA guidelines, when all other interventions are contraindicated in severe aortoiliac disease, the only indicated surgery is axillofemoral bypass [12].

This case also had ethical implications in terms of maternal well-being. The ACC/AHA identified multiple risk factors for amputation in patients with chronic limb-threatening ischemia [12]. Of these, our patient had one: ischemic pain at rest. Our patient affirmed multiple times that she desired amputation over loss of pregnancy. However, not all women may have the same goals of care. Overall, patient compliance with the recommended treatment is essential. In this case, noncompliance with anticoagulation prior to pregnancy may have led to increased thrombotic burden. Cocaine use also likely led to increased vasoconstriction which resulted in increased symptoms. Lack of compliance can be due to a multitude of factors associated with numerous health system constraints, including lack of supportive resources during pregnancy and potential loss of the mother's health insurance after birth. Resources are currently being examined at a national level to address the care of women after pregnancy [15].

Management of pregnant patients with arterial occlusive disease and previous abdominal vascular grafts is challenging due to the demands of the gravid uterus competing for collateral vasculature. Limited surgical access to the area due to the gravid uterus can lead to potentially devastating outcomes, including loss of limb and/or the fetus. A multidisciplinary approach is required to balance the complex medical management of these patients, along with clarifying maternal goals of care. Conservative management is generally recommended with anticoagulation and plan for surgical intervention only in cases of true limb-threatening acute ischemia. In this patient, symptoms failed to improve after pregnancy and redo aorto-iliofemoral reconstruction was performed after delivery when the uterus was no longer enlarged and engorged.

## Additional Points

*Teaching Points.* (1) Motor function deficits can help guide the need for intervention in a patient with complex peripheral arterial disease. (2) Timing of delivery in a pregnancy complicated by peripheral artery disease and bypass grafts needs to be individualized. (3) A multidisciplinary team approach including vascular surgery, hematology, maternal fetal medicine, anesthesiology, and neonatology is recommended to optimize both maternal and neonatal outcomes.
